# Clinicians’ views of prescribing oral and intravenous bisphosphonates for osteoporosis: a qualitative study

**DOI:** 10.1186/s12891-023-06865-1

**Published:** 2023-09-29

**Authors:** Simon Bishop, Melanie Jay Narayanasamy, Zoe Paskins, Nadia Corp, Anastasios Bastounis, Jill Griffin, Neil Gittoes, Jo Leonardi-Bee, Tessa Langley, Opinder Sahota

**Affiliations:** 1 Nottingham University Business School, University of Nottingham, Nottingham, NG5 1PB UK; 2 School of Medicine, University of Nottingham, QMC Campus, Nottingham, NG7 2UH UK; 3 School of Medicine, Keele University, David Weatherall Building, Newcastle-Under-Lyme, UK; 4 ST5 5BG and Haywood Academic Rheumatology Centre, Haywood Hospital, High Lane, Burslem, Stoke-On-Trent, ST6 7AG UK; 5 School of Primary, Community and Social Care, Keele University, Newcastle-Under-Lyme, ST5 5BG UK; 6 Division of Epidemiology & Public Health, School of Medicine, University of Nottingham, City Hospital, Nottingham, NG5 1PB UK; 7 Royal Osteoporosis Society (ROS), St James House, The Square, Lower Bristol Road, Bath, BA2 3BH UK; 8 Institute of Metabolism and Systems Research, University of Birmingham, Birmingham, UK; 9 Faculty of Medicine & Health Sciences, University of Nottingham, Nottingham, NG5 1PB UK; 10 Department of Healthcare of Older People, Nottingham University Hospitals NHS Trust, Nottingham, NG72UH UK

**Keywords:** Bisphosphonate regimens, Adherence, Zoledronate, Qualitative research, Treatment choice

## Abstract

**Background:**

Bisphosphonate medications, including alendronate, ibandronate and risedronate administered orally and zoledronate, administered intravenously, are commonly prescribed for the treatment of osteoporosis based on evidence that, correctly taken, bisphosphonates can improve bone strength and lead to a reduction in the risk of fragility fractures. However, it is currently unclear how decisions to select between bisphosphonate regimens, including intravenous regimen, are made in practice and how clinicians support patients with different treatments.

**Methods:**

This was an interpretivist qualitative study. 23 semi-structured telephone interviews were conducted with a sample of general practitioners (GPs), secondary care clinicians, specialist experts as well as those providing and leading novel treatments including participants from a community intravenous (IV) zoledronate service. Data analysis was undertaken through a process of iterative categorisation.

**Results:**

The results report clinicians varying experiences of making treatment choices, as well as wider aspects of osteoporosis care. Secondary care and specialist clinicians conveyed some confidence in making treatment choices including on selecting IV treatment. This was aided by access to diagnostic testing and medication expertise. In contrast GPs reported a number of challenges in prescribing bisphosphonate medications for osteoporosis and uncertainty about treatment choice. Results also highlight how administering IV zoledronate was seen as an opportunity to engage in broader care practices.

**Conclusion:**

Approaches to making treatment decisions and supporting patients when prescribing bisphosphonates for osteoporosis vary in practice. This study points to the need to co-ordinate osteoporosis treatment and care across different care providers.

**Supplementary Information:**

The online version contains supplementary material available at 10.1186/s12891-023-06865-1.

## Background

Bisphosphonate medications are routinely prescribed for the treatment of osteoporosis, based on evidence that, correctly taken, they can improve bone strength and lead to a reduction in the risk of fragility fractures [1, 2]. Bisphosphonates including alendronate, ibandronate and risedronate administered orally and zoledronate, administered intravenously, are commonly in use, with each being shown to reduce the risk of fragility fractures [3]. Weekly oral alendronate is the first line treatment in several countries, including the UK. However, the adherence to oral medication has frequently been found to be poor, resulting in preventable fractures [4, 5]. A recent systematic review suggested that persistence with oral bisphosphonates two years after initiation on the medication ranged between 12.9% and 60.6% [6], varying by age, frequency of dosing, and factors associated with the wider healthcare system. Lack of persistence severely blunts the efficacy of medication in reducing fracture risk [7]. Potential reasons for low adherence to oral bisphosphonates include relatively complex dosing instructions and common side effects [8]. Patients taking weekly alendronate are advised to take it in the morning on an empty stomach, with a full glass of water. Patients should then remain upright, fast and avoid other oral medication for half an hour after ingestion [9]. These directions are to counter the upper gastrointestinal irritation which can be experienced and to maximise the bioavailability of the drug. Further reasons for low adherence include patient perceptions that the benefits of bisphosphonates are unclear, and wide-ranging concerns and uncertainty about safety and necessity for treatment [10].

Given patient concerns and low adherence to oral bisphosphonates, zoledronate administered intravenously, usually prescribed at 12-month intervals, provides an alternative option. A recent systematic review and network meta-analysis suggested zoledronate may reduce the risk of fracture more than other bisphosphonates [3]. As this focused on randomised control trials, in which drug taking is closely monitored, this may indicate that the reduced risk came at least in part from the drug action itself, although other factors such as adherence cannot be completely discounted. Anecdotal evidence suggests zoledronate is often prescribed when it is judged that adherence to oral bisphosphonates is likely to be poor, and patient accounts suggest that there is variation in when, and in what patient circumstances, zoledronate is preferred [11]. Current research does not include exploration of how such decisions are made in practice. In addition, some of the complexities in prescribing bisphosphonates relate to all forms of the drug and may increase uncertainty around treatment decisions. These include the possibility of rare severe side effects and associated decisions over treatment duration. Rare, recognised side effects include osteonecrosis of the jaw [12] and atypical femoral fractures [13]. Estimates of the prevalence of both of these side effects vary, although in both cases the risk is greater with increased duration of exposure to bisphosphonates. Considering this, a ‘treatment pause’ after 3 to 5 years of treatment has been supported [14]. Although bisphosphonates have been found to reduce the relative risk of hip and vertebrae fracture by between 40%-70% [15], outweighing the risks of severe side effects [16], the way that prescription complexities shape treatment decisions and care in practice remains unclear. For example, a UK study of GPs’ and Pharmacists’ experiences of prescribing bisphosphonates suggested that there was some awareness of osteonecrosis of the jaw but also uncertainty on how to manage the risk of the condition, as well as the risk of other co-morbidities [17]. A qualitative study of polypharmacy prescribing amongst GPs in New Zealand suggested variations in opinions about when bisphosphonates should be discontinued when prescribed alongside other medication [18].

A recent systematic review of studies into the acceptability of bisphosphonates among stakeholders, including clinicians, suggested that clinicians themselves had some uncertainty over the potential benefits of bisphosphonates when considered against potential concerns [10]. Individual research studies in Canada [19] and Australia [20] have suggested that primary care physicians vary considerably in their understanding of medication for osteoporosis and some report a great deal of confusion and uncertainty over medication choice and duration, and also do not feel well supported by diagnostic tests such as bone density scans [19]. Existing work has identified how a variety of factors may play into treatment decisions for osteoporosis in practice, including patient preferences [21], the relevance of guidelines [19] the availability of scan results [22], and in some contexts the costs of the medication for the patients or the wider health system [20, 23]. However, there has to date been little qualitative research on the particular issue of selecting between intravenous and oral medication and supporting patients on different forms of treatment. Therefore, the current study investigates clinicians’ experiences of prescribing different forms of bisphosphonate medication, focusing on how treatment decisions are made in practice and how this relates to ongoing processes of treatment and care.

## Methods

This study formed one work package within the BLAST OFF (Bisphosphonate ALternAtive regimenS for the prevention of Osteoporotic Fragility Fractures) study, funded by the UK NIHR HTA programme. The aim of this work package was to explore clinicians’ and clinical experts’ experiences of using different bisphosphonate regimens, and understand their preferences for, and processes of engaging with, alternative bisphosphonate regimens compared to alendronate. Ethical approval was obtained from the North West- Preston Research Ethics Committee (REF: 19/NW/0714). Semi structured interviews were conducted over two periods: June 2020- August 2020, and March 2021.

This was an interpretivist qualitative interview study, with this approach taken in order to understand clinicians’ experiences of treating and managing osteoporosis within the contexts of their everyday work. This took an inductive approach, with the focus on explaining the accounts of clinicians in their own terms, rather than testing prior theory. A sample of GPs, secondary care clinicians, specialist experts (including those involved in research), as well as those providing and leading novel treatments were recruited for qualitative interview. These groups were purposefully sampled to include those with a good knowledge of the bisphosphonate regimens in use and involved the following approaches. Firstly, GPs were contacted through a snowball approach beginning with the existing professional networks of the study team. During covid-19 related restrictions on recruitment, existing networks of the study team allowed the identification of GPs both with and without specialist/research involvement and commissioning/service leadership for osteoporosis and bisphosphonate treatment. Study team members identified potential participants, and a research advertisement was also placed in the West Midlands CLRN newsletter for research active GPs. GPs who were interested in taking part were invited to contact the study team and were then sent a Study Information Pack which included an invitation letter/email and Participant Information Sheet. Secondly, the research team contacted specialist clinicians, including those involved in research and service leadership. These respondents were identified through snowball sampling beginning with the study team. Eligible individuals identified by the study team were sent the Study Information Pack. Thirdly, the research team sampled from two specific areas where different or novel first line bisphosphonate regimens are used. This included a service giving intravenous zoledronate treatment first line in people’s homes, and a service with a programme of blood test monitoring which is not usual practice elsewhere in the UK. Again, eligible individuals identified by the study team were sent the Study Information Pack.

### Data collection

Interview schedules for participants were developed in collaboration with the study team and steering group, which included Patient and Public Involvement and Engagement (PPIE) representatives and comprised questions about clinicians’ experiences of managing patients with osteoporosis, perceptions about providing bisphosphonate treatment), and general clinician and service factors; the interview schedule is included in the supplementary material. All participants provided informed consent and all interviews were conducted over the telephone.

### Data analysis

Data analysis took an interpretivist approach [24], undertaken through a process of iterative categorization [25]. This which was used to provide a clear trail of the development of analytic themes from the initial coding of the data. All interviews were recorded digitally, transcribed verbatim and anonymised. The qualitative analysis program NVivo (version 12) was used to support open coding of interview data, which helped to identify key ideas and emerging issues, which NVivo describes as “nodes”. The first five transcripts were independently coded by two researchers with social science backgrounds, and the coding was then discussed and compared, which enabled data interpretations to be reviewed and refined as appropriate. Additional sub-nodes were agreed by the two researchers, ensuring that other relevant issues were captured and categorised. Remaining transcripts were then shared between the two researchers to be coded based on the agreed coding framework. Following coding of all the transcripts, the researchers considered which nodes were most pertinent to the research question, and these became the focus for the next stage of the analysis. These nodes were systematically re-read and patterns of data were identified, considered alongside the research questions and previous literature, with interpretive notes added. Potential explanations for the different responses of study participants were considered between the two researchers. This provided a first point for critical reflexivity; both researchers had also been involved in analysing qualitative data from osteoporosis patients many of whom reported significant challenges with treatment and assumptions being made of the clinicians’ data was discussed, along with contradictory data. Preliminary explanations for the way participants in different roles described the issue were then presented to the wider study team, which included clinicians with experience of providing specialist and primary care osteoporosis care. This provided a second point for critical reflexivity, with the plausibility, strength and relevance of emerging explanations considered. The results below firstly cover how the clinicians chose between alternative forms of treatment, and secondly address issues of support and engagement.

## Results

In total, 23 clinicians took part in semi-structured interviews. This included 9 general practitioners from across England and 11 secondary care clinicians, including 8 consultants (working in either General Medicine, Endocrinology, Metabolic Medicine, or Orthogeriatric Medicine) and 3 specialist osteoporosis nurses, with two of the latter having experience in prescribing (see Table 1 below). All secondary care clinicians worked across the Midlands or the Yorkshire and the Humber region in hospital settings, which included bone clinics, triage referral centres, and fracture liaison services. We also interviewed three nurses providing a community-based IV zoledronate treatment in the English Midlands. The duration of the interviews ranged from between 20 and 60 min.

**Table 1:** Table of study participants

Clinician stakeholder group	Total number of interviewees	Location(s)	Specific services	Specific roles
General Practitioners	9	West Midlands (*n* = 5) Northeast England (*n *= 2) Southeast England (*n* = 1) East Midlands (*n* = 1)	General Practice (*n* = 8) Single Point of Access service (*n* = 1)	GP Partner (*n* = 5) Salaried GP (*n* = 4) Osteoporosis/musculoskeletal specialist roles (*n* = 2)
Secondary Care Clinicians and service specialists	10	East Midlands (*n* = 7) West Midlands (*n* = 3)	Secondary care bone specialist services e.g. Bone clinics and Fracture Liaison Services (*n* = 10)	Consultants (*n* = 7) Specialist nurses (*n* = 3)
Providers of novel treatments	4	Midlands (*n* = 3) Yorkshire and the Humber (*n* = 1)	Community nursing service (*n* = 3) Secondary care bone specialist service	Nursing lead and nursing team members (*n* = 3) Consultant (*n* = 1)

Overall, prescribing bisphosphonates was seen not to be a straightforward matter, with treatment choice, patient adherence and support being identified as key challenges. The sections below cover these issues in turn, highlighting differences in responses between the participant groups.

### Treatment choice

All participants were aware of NICE guidelines identifying oral alendronate as the first line treatment for osteoporosis and identified alendronate as the most frequently prescribed medication. However, there were differences between secondary care specialists and GPs in how they made choices to deviate from this and consider alternative treatments. Oral risedronate, oral liquid and dispersible (Binosto)—as opposed to tablet—alendronate, Denosumab or intravenous (IV) zoledronate were mentioned most frequently as alternatives. Secondary care specialist clinicians reported comparative confidence on how to make treatment decisions with respect to a range of factors included existing co-morbidities, potential medication interactions, response to first line treatment in terms of expected impact on bone density, as well as side effects and adherence. As part of this, they commonly described how their decisions would be informed by specific issues including patient creatine level, renal function, digestion, bone strength, cognitive function, and jaw osteonecrosis.

*“So on the ward essentially we start with alendronic acid and people who have GI side effects which we know, we sometimes use risedronate or Binosto.”* (B019c_Consultant)

As one element of this, secondary care clinicians also reported comparative confident in making the choice between oral and intravenous bisphosphonates, predominantly based on the extent to which it was felt patients would cope with or adhere to oral treatment, but also based on their response to oral treatment, examined through follow -up tests for bone health.

*“if I perceive that they're unlikely to take oral medication properly, then my conversation would be more towards encouraging them to go for intravenous, but if it is someone who well educated, they know about both, then I give them a choice – this is oral – this is intravenous – you have this once weekly – you have this once yearly or once every eighteen months”* (B002c_Consultant)

*“*[rheumatology consultants] *sort of say ‘oh it’s not working, they're not taking it anymore, can we ‘ZOL’ them’ or whatever and then I just take it from there.”* (B003c_Specialist nurse)

Secondary care clinicians related their confidence to make treatment decisions, including to ‘ZOL’ patients (start them on a course of intravenous zoledronate), to several factors. These included access to relevant specialist expertise, the regularity with which such decisions were made, their ability to directly access and interpret diagnostic tests, controlling the monitoring and follow up of the patients, and adjusting the treatment as deemed appropriate in light of this routine. Some, but not all, of the secondary care specialists also introduced the principle of patient choice of treatment and/or patient centred care on how such decisions were made.

*“when I diagnose anybody and start talking about treatments, I always talk to them about both [oral and IV treatments]”* (B011c_Specialist nurse)

*“We try and follow the NICE guidelines if we can but that’s not a firm rule. It’s very individualised to the patient.”* (B004c_Consultant)

A similar picture was presented by the community team within the novel service administering IV zoledronate as first line treatment. In this service, treatment decisions were initially made by secondary care consultants, with the community team reporting that they remained involved in ongoing treatment choice and duration, and commonly requested bone density or blood marker tests. This allowed them to continue to consider which treatments were appropriate for the patients and gave them confidence to make decisions.

In contrast, most of the GPs conveyed a higher degree of uncertainty around treatment choice including the benefits of treatment for particular patients as well as treatment duration.

*“Sometimes if people are in the middle of a course of dental treatment I'm unsure [ … ] whether I should be starting a Bisphosphonate or not”.* (B006c_GP)

*“And then we never really know whether to stop [bisphosphonates] or not. Each of the guidance is different, isn't it, the NICE side and the NOGG.* (B010c_GP)

Several reasons were suggested for this uncertainty, including conflicting guidance, potential medication interactions, complications and side effects, and patients’ reactions or engagement with treatment.

*“I guess part of the problem is that we have a lot more information in our health records and we have a prescribing system that, you know, it brings up every single side effect and interaction that ever lived … you're sitting there thinking “should I be worried about this or not”* (B009c_GP)

*“So, there’s sort of this, I have to say the Guidelines, although they’re quite clear and on the website, obviously patients don’t fit into Guidelines”.* (B018c_GP)

While two of the GPs in the study identified a specialist interest in osteoporosis, most suggested that they relied on the advice from specialist services to help them make decisions, and would only deviate from the first line treatment (oral alendronate) following consultation with secondary care specialists. This included decisions to refer patients for consideration of the intravenous regimen whichGPs felt they could not offer independently. In some instances, GPs identified strong and helpful advice from secondary care specialists, while in other instances this was seen as absent or more difficult to access.

*“We've got a very good osteoporosis service near us and if I'm honest, they're that good that we don't have to do very much in the way of work really,* [...] *we get a letter saying ‘x, y and z, this is their FRAX* [Fracture Risk Assessment] *score and please prescribe”* (B009c_GP)

*“in [Place 1] they report your DXA scan … They give you the numbers, they will tell you whether it’s Osteopenia, Osteoporosis, or normal … But in [Place 2] they will actually then back that up with advice, based on what the information the Clinician is given, to sort of make some recommendations about treatments … which is really, really, helpful. If I could have one thing, I would have that kind of a system in [Place 1]”.* (B015c_GP)

As this suggests, not all GPs were aware of how to act on assessments and diagnostic tests.

*we had a letter saying please do a FRAX risk assessment in something, and I was thinking well OK, I don’t know what a FRAX risk assessment is, but I’ll Google for it and found it, and did it. And then I couldn’t understand why, what I should then do as a result of it”.* (B008c_GP)

*“sometimes I might not know what to do with a* [diagnostic test] *result if it’s a bit difficult … I’d have to ask advice … ring up the bone density team, speak to the consultant, just say ‘I'm sorry, I don't know what to do about this’.”* (B006c_GP).

In certain circumstances, GPs stated they did seek to move patients to alternative medications after alendronate treatment had been tried. GPs described a process of trial and error on which treatments to take, responding to issues as they arose and in most cases referring patients back to specialist secondary care services.

*“*[alternative treatment would be considered if] *severe side effects really or it hadn't worked, the patient had got further fragility fractures. And I’d probably seek guidance again, specialist guidance about that before I prescribed it”. *(B006c_GP)

Only one GP suggested they directly prescribed IV zoledronate to patients, while a further GP suggested they counselled patients on IV zoledronate as an option, but would not prescribe it themselves as it would be administered by specialist services. Other GPs stated they were aware that patients were receiving IV zoledronate in secondary care, but generally they did not get involved in supporting this treatment.

### Patient adherence and support

Closely tied to issues of treatment choice were the participants’ experiences of patient adherence to treatment and the ways in which clinicians felt they could support this through their actions and services. As a common experience across participant groups, it was suggested that it was very usual for patients to resist, avoid, or forget to take oral bisphosphonate medication. Central to this was the suggestion across participants that oral bisphosphonates are comparatively burdensome for patients due to complex instructions for taking the tablets, and frequent side effects, while the benefits are comparatively opaque.

*Because you are having it once a week it’s easy to forget it. So, you know, adherence is one of the issues.”* (B017c_Consultant)

*I’ve got one lady who restarted it three times and she doesn’t take it. So she’s not interested actually* (B010c_GP)

*“compared to other tablets, it’s a common one that I think patients decide not to take and don’t tell us about it”.* (B005c_GP)

It was therefore seen as challenging to articulate the potential benefit of a reduction to longer term fracture risk against the effort, inconvenience and side effects of taking the medication, with respondents commonly stating that it is difficult for patients themselves to assess the success of treatments.

*“Obviously as time goes by people are taking medication for something which is an asymptomatic condition and so generally sort of drifting off treatment”* [B023c_Consultant]

*‘no-one ever thanks you for not getting a fracture, do they, do you know what I mean?”* (B009c_GP)

For IV bisphosphonates, adherence was seen as less of an issue. However, participants did suggest that acute side effects could lead patients to avoid treatment and in certain cases patients were nervous about receiving what was seen as a higher or stronger dose of medication. More commonly, it was suggested certain patients were nervous about receiving IV treatment, for reasons such as coming into hospital or a fear of needles or concern about the higher strength of dose to be received.

*“a lot of people say ‘well if I’m going to have side effects, you can’t wash it out. If I have a tablet I can stop it’* (B012c_Specialist nurse)

In view of patient concerns about bisphosphonates, participants described various communication tactics they adopted within patient consultations to try to encourage adherence. These were quite varied and differed between individuals, but tactics included coaching patients on instructions for taking medication verbally and/or in writing, framing the medication positively around improving bone strength, setting expectation, emphasising the potential negative consequences of fracture and encouraging patients to interpret ‘no news as good news’.

*“you might have had a stumble, and the fact that they’ve not actually fractured shows you that the medication well may be benefiting.”* (B020c_Community nurse)

*“*[bone density scans] *can sometimes be quite a powerful message for people in terms of actually ‘look, you’re five years older but actually your bone health is either better or the same as it was five years ago”.* (B009c_GP)

In addition to these tactics, participants from each group identified the importance of regular follow up, supplemented by bone density diagnostic testing to support patients and demonstrate to patients that the medication was ‘working’ to improve bone strength. Secondary care clinicians generally identified that they had systems in place for such follow up. For patients taking oral bisphosphonates this would usually be for a period after treatment initiation, and for IV bisphosphonates this took place alongside the infusions.

*“I normally like to see them once again in my clinic to make sure they’re taking it and so on before I discharge them back to the GP. Research has shown that, you know, some form of follow up does improve compliance as well.”* (B004c_Consultant)

*“The nurses follow them up regularly to do the infusion. After three infusions, we will see them, look at the DXA to see whether they need to remain a bit longer on the Bisphosphonates.”* (B002c_Consultant)

A similar picture was presented by the clinician in the study representing the service which involved monitoring blood markers for bone health at commencement and at six months. The test was described as more sensitive in terms of its ability to identify change compared with bone density scanning, as well as less burdensome for patients. The test was also seen as providing a structure for follow up and support.

*“I think because people like to know that someone’s checking on them and they’re getting a bit of feedback and if they’re going to be asked to take the medication that somebody is checking that it’s working, so they’re not very burdensome to have a blood test and a phone call, definitely less burdensome than coming and having a bone density scan*”. (B023c_Consultant)

In comparison to secondary care clinicians, GPs generally suggested that supporting patients with medication presented a number of challenges. This included the limited resources, time and competing priorities which meant that focusing on osteoporosis was difficult.

*“So you’ve got ten minutes to explain to someone that their bones are thinning, they’re at risk of fractures and I’m putting you on this tablet and this is how you have to take it.* (B005c_GP)

*“So with her, what I ended up doing is saying ‘look, would you be willing to try it and I’ll ring you in a month, I’ll see how you’re getting on … I haven’t got the time to do that with every patient,”* (B005c_GP)

In contrast to the specialist secondary care clinicians with services directed towards osteoporosis, GPs described the way in which osteoporosis was frequently just one of a number of their patients’ co-morbidities, and one which often did not take priority during consultations. For this reason, there was a tendency for support with osteoporosis medication to ‘get lost’ in the pressure to provide other forms of care. As an exception, one GP described themselves as having specialist interest in osteoporosis and had established an audited system to routinely follow up patients at three weeks following the initial prescription. However, the rest of the GPs in the study suggested they had no standard system or structured follow up to review medications or try to increase adherence.

*“Well if we had infinite resources then I would have a sort of annual review* [of patients] *in general practice but that’s never going to happen because GPs are just too busy at the moment”.*(B006c_GP)

*Well it’s not possible for GPs to be checking on people every three months to see if they’re taking it”.* (B010c_GP)

*“I think we’re as I’ve already discussed, not good at picking up when compliance drops off. Because actually we haven’t got a formal strategy for auditing or reviewing”.* (B016c_GP)

One GP suggested a lack of financial incentives as a barrier to this.

*As far as I know osteoporosis isn’t a QOF* [additional payment targeted to the treatment of certain conditions] *thing so there’s no incentive there to follow up”.* (B010c_GP)

As with treatment choice, some GPs reported strong support and advice from secondary care specialist services, which helped them engage patients with treatment, others suggested that services were lacking in their area or that access was difficult.

*“Yeah, I think there’s a bit of a gap in that Secondary Care consultants don’t really understand what it’s like working in Primary Care* (B006c_GP)

As this last quote illustrates, some GPs focused on the challenge of managing treatment for osteoporosis in the ‘reality’ of primary care work.

For the clinicians providing IV zoledronate in the community, structured follow-up was seen as a strength of the service. Visiting patients in their home was seen as providing an opportunity to discuss issues relating to the treatment, identifying additional care needs that could lead to further referrals and assessments and was generally seen as a chance to provide personalised care.

*“your attention is a hundred percent on that person or the people that are in the room with you* (B022c_Community nurse)

*whilst you’re in somebody’s home you can make assessments of, you know, sort of, environment as well”.* (B021c_Communiy nurse)

In comparison with GPs, this group described the benefits of running a ‘bespoke’ form of service focused on treating osteoporosis. This was also seen as having other benefits, including the ability to span the boundary between the specialist teams located in acute care and community and primary care settings. A handful of administrative issues were identified by the team, particularly in organising visits to patient homes during Covid-19 restrictions. However, in general, participants providing IV zoledronate in the community felt that they were well able to support patients taking the medication and work through challenges on an individual basis.

## Discussion

Overall, the findings of the study have shown the different experiences of clinicians prescribing and supporting patients on alternative forms of bisphosphonate medication. One way of interpreting the findings is that for secondary care clinicians and those providing specialist services, treatment and care for osteoporosis was seen as ‘complicated’, needing to take into account a number of patient level factors and requiring appropriate forms of follow up care. However, the choice and processes of treatment was largely seen as under their own control and the schedule of follow up could be set as they saw appropriate, with participants identifying schedules and processes for checking up with patients after they had started on a course of medication. This does not necessarily imply that all patients under the care of secondary care specialists received or attend follow up appointments and tests, and indeed a number of participants suggested it was common for patients to ‘drop off’ systems for follow up. Nevertheless, secondary care specialists conveyed confidence that they could make appropriate treatment decisions for patients, including around identifying patients that they felt would benefit from intravenous zoledronate.

In contrast to the above, for most of the GPs in the study, osteoporosis care was ‘complex’ [26] with no clear pathway or approach to decision-making identified, and multiple overlapping priorities and concerns shaping their decisions. This could be seen as stemming from their varying access to specialist expertise as well as wider assessments and diagnostic tests. It could also be seen as arising from their need to balance osteoporosis care with patients’ wider care needs including co-morbidities, divergent preferences, values, and beliefs, changing circumstances and ongoing (non) engagement with treatment. Previous studies of clinicians’ views of osteoporosis have similarly identified the challenge primary care physicians have in treatment choice and supporting patients with medication [19, 20]. Previous qualitative studies of GPs in Australia [20, 27], the UK [22], France [28] and Canada [19] have identified uncertainty and a lack of confidence around certain elements of the diagnosis and treatment for osteoporosis, including the use and interpretation of assessment and diagnosis tools and their own role in the care pathway. These studies also highlight the competing priorities, time and resource constraints of GPs to prioritise bone health when managing multiple co-morbidities and the need for guidance that is appropriate to the challenges of primary care.

Our findings add to this evidence and suggest clinicians working in primary care require consistent guidance on treatment decisions, access to specialist expertise, as well as capacity and resources to provide appropriate forms of follow up care. At the same time, our study points to the way that knowledge and resources for managing osteoporosis are distributed across the healthcare system. Indeed, a number of GPs saw their involvement as one part of a wider health care system and noted the importance of support from secondary care specialists, albeit with varied views of how accessible these were in practice. In some instances, GPs reported good support from Fracture Liaison Services within their area, although these were not universally present.

The group of clinicians providing the novel service for IV zoledronate as first line treatment had a particularly favourable opinion of the care they provided in terms of being both tied into secondary care specialist expertise and able to provide ‘holistic’ care within community care settings. Indeed, whether provided in domestic settings or in acute care, the schedule of appointments to administer IV bisphosphonates was seen as providing a structure for following up patients and for example a chance to book relevant diagnostic tests. This was also seen as a positive factor in the service monitoring blood markers for bone health as standard care.

The findings of the current study can in some ways be seen to mirror the findings of previous studies on patients’ experiences of osteoporosis treatment. At the general level, previous studies have reported the concerns and perceived burden of adhering to bisphosphonate regimen [10, 11]. With direct relevance, a study of members of a national osteoporosis patient group in Canada found that patients commonly perceived that primary care physicians lacked interest in their bone health, in comparison to those providing specialist bone services [29]. While this is perhaps not surprising, the current study provides insight into the way intricacies of treatment options may play into patients’ perceptions, with a lack of confidence to make treatment decisions in primary care potentially experienced by patients as a lack of interest or concern.

It should also be noted that the context of the current study is the English NHS, which is a publicly funded system. While previous studies have reported professionals’ concern over the affordability of treatment options to patients [20, 23], this was not regularly brought up by the participants here, although cost and benefit is certainly taken into account in the development of national guidelines which inform clinicians’ decision making. The Quality and Outcomes Framework (QOF) incentivises primary care to identify people with osteoporosis, but not to support ongoing management such as adherence. One GP in the current study suggested that this lack of QOF incentive meant that ongoing management was less of a priority for them, and performance against the osteoporosis QOF is currently lower than for other long-term conditions.

Building on the above points, the study highlights the importance of co-ordinating bone health care across primary and specialist providers to allow each to play to the strengths of their own positions. Professionals working in different roles have access to different knowledge and resources, as well as different opportunities to engage with patients around management and treatment of osteoporosis. Given the limited time and resources to focus on bone health within general practice, the consistent availability of specialist services would appear to be of central importance. At the same time, and reflecting on how care across different professionals is experienced by patients [29], consideration should be given to appropriate ways of discussing the way responsibility for their care is shared.

The strength of this study is that it includes the views of healthcare professionals working in different parts of the healthcare system, around the specific issue of alternative forms of treatment for osteoporosis. However, the study also has a number of limitations. The study was limited to a purposeful sample of clinicians who were willing to take part in the study during a period of Covid-19 restrictions and associated pressure on the health service. This may have affected the nature of the accounts provided, which are understood to reflect the interests and knowledge of the research participants, and the findings are not directly generalisable to the general population of primary and specialist osteoporosis clinicians. In addition, the sample was not stratified to include participants from regional healthcare systems with particular characteristics, and no wider data was included on the contexts of the clinicians’ work. It is therefore not possible to examine here how work or health system contexts shaped the experiences reported here, beyond the individual accounts provided by the clinicians. A further limitation is that it is only the clinicians’ view that is examined here; other work packages in the Blast Off study have examined the experiences of patients receiving different forms of osteoporosis treatment, and future work may usefully bring the patient and clinicians experiences together for further analysis.

## Conclusion

There is considerable variation in how bisphosphonate treatment choices are made in practice, as well as in the level of follow up and support. A recent systematic review has suggested that IV zoledronate is more effective at preventing fragility fractures than other bisphosphonates [3]. The current study may suggest that at least part of the reason for this could be that clinicians utilise IV appointments to take on aspects of osteoporosis care. Future research could therefore seek to distinguish between the effects of the IV zoledronate medication itself, issues of adherence and the ‘value added’ of personal support which surrounds the medication administration. Further research could also usefully focus on interventions to improve the access to specialist osteoporosis knowledge within primary care.

### Supplementary Information

**Additional file 1.** Blast-Off interview questions.

## Data Availability

The dataset generated and analysed during the current study are not publicly available as they may contain information that could compromise research participant privacy, but may be made available from the corresponding author on reasonable request.
