# Supplementary material for: Clinicians’ views of prescribing oral and intravenous bisphosphonates for osteoporosis: a qualitative study
Source: BMC Musculoskelet Disord. 2023 Sep 29;24:770. doi: 10.1186/s12891-023-06865-1 (PMC10540377; doi:10.1186/s12891-023-06865-1)
Supplement: Supplementary file 1 — Additional file 1. Blast-Off interview questions. [file 12891_2023_6865_MOESM1_ESM.docx]

**Blast-Off interview questions –Clinicians joined**

**Part 1 - Introduction and familiarisation**

- Firstly, could you give me an overview of your professional role and this service? What tasks and duties come under your responsibility?

[Researchers to nuance this as appropriate; aim is to understand general nature of respondents work role/responsibilities and therefore how treatment for osteoporosis/bisphosphonate medications fits within this]

**Part 2 - Bisphosphonate treatment**

You have been invited to participate in the study because you have been identified as someone potentially involved with the provision of bisphosphonate treatments, for example (Alendronate, Ibandronate etc.)

Service process

- Could you tell me about the bisphosphonate medicines or treatments which you are involved with prescribing/administering for patients with osteoporosis?
- How often are you involved in prescribing/administering such treatments?
- What are your specific roles and responsibilities around this? How does this compare with other healthcare professionals?
- Could you talk me through the process of prescribing/administering these treatments? [Potential prompts]
- Do you carry out any tests, e.g. blood tests, prior to infusion? For what reason?
- What are the main reasons that patients would be given IV Bisphosphonates? [Potential prompts]
- What are the main reasons that patients are referred to you from primary care?
- Key differences between oral and IV Bisphosphonates e.g. Administration; Side effects associated with oral Bisphosphonates
- **[Secondary care]** How do you make decisions about who is likely to benefit from treatment and/or who to accept once a referral to secondary care is made?
- **[Primary care]** How are patients identified? How do you make decisions about who is likely to benefit from medicine/ treatment?
- Do you ever consider a Bisphosphonate and then decide that it is not in the patient’s best interest? Can you describe? To what extent do you involve the patient in this discussion?
- What issues do you consider when making a Bisphosphonate choice/ recommendation? [Potential prompts]
- How do you decide which specific Bisphosphonate medication to prescribe? Are you directed by guidelines; management; other factors?
- Nature of the patient’s referral to secondary care
- Patient capacity
- Dental health
- Kidney function
- Patient choice
- Multi-morbidity
- Do you carry out any tests beforehand? For what reason?
- What is the length of time that you prescribe a course of Bisphosphonate medication for?
- Do you ever feel uncertain about recommending a Bisphosphonate? When? Why?
- Do you ever feel uncertain about managing patients on Bisphosphonates? [Possible prompts]
- (If clinician is not a specialist in Osteoporosis, we can explore whether this is a factor that undermines confidence)
- Do you ever signpost patients to other help and resources? E.g. Royal Osteoporosis Society?
- Do you yourself use other help and resources to support you in your care of these patients? [Possible prompts]
- Do you ever use specialist helplines/ websites/ services to get information, advice or resources? Or consult with specific colleagues?
- [**Secondary care clinicians**] What do you think are the main issues that should be considered when primary care clinicians make a secondary care referral to your service/ department?
- **[Primary care]** What do you think are the main barriers and facilitators to making secondary care referrals?
- **[Primary care]** What issues do you consider when making a secondary care referral? What are the barriers and facilitators to this?
- What information and instructions are patients given in order to take the medicines and by whom? [Possible prompts]
- To what extent do you think that patients are clear about what Bisphosphonate medication does (and doesn’t do)? E.g. does not relieve pain
- Are there particular instructions that you prefer to leave to a pharmacist or that you feel are better explained in an information leaflet?
- Do you have to give patients any particular information and instructions regarding the IV treatment? [Possible prompts]
- To what extent do you think that patients are clear about what Bisphosphonate treatment does (and doesn’t do)? E.g. does not relieve pain
- Are there particular instructions that you prefer to leave for other members of your team/ other healthcare professionals or via resources such as leaflets?
- To what extent do you think that the instructions that patients receive are effective? Why? Why not?
- Do you find that there are any particular instructions regarding Bisphosphonates which patients struggle to adhere to? Which ones? Why do you think this is? [Possible prompts]
  - **[Specific instructions for administering medication]** e.g. Take on empty stomach; Take with full glass of water; Stand or sit upright for 30 minutes after taking them; Wait between 30 minutes and 2 hours before eating food or drinking other fluids
  - **[Wider follow up and self-care during treatment]** e.g. Looking after teeth and having regular dental check-ups; Getting enough Calcium and Vitamin D; Drinking enough fluids before and after infusion; Healthy eating; Exercise; Reducing alcohol intake; Stopping smoking
- How well do you think patients adhere to such instructions?
- Do you follow up patients on these medications and treatments? Could this be improved? [Possible prompts]
- Are patients offered DXA scans or bone marker tests? Or do patients request these themselves?
- Are there any variations in the Bisphosphonate treatments that you offer? What accounts for this variation?

Service management and barriers

- What, if any, are the key challenges or barriers in providing the medications/treatment pathways you have described? [Possible prompts]:
  - Identifying patients
  - Patient attendance
  - Communicating with patients
  - Patient understanding
  - Patient values and beliefs
  - Patient lifestyle/ self-care
  - Side effects
  - Changing patient health status/ patient co-morbidities
  - Clinician work/ time pressures/ training (potentially impacting on assessment/ decision-making/ prioritisation)

- How do you think such challenges and barriers can be overcome?

- In your view, how well do your patients persist Bisphosphonate treatment over the long term?
- What, if any, do you think are the key barriers to treatment adherence and persistence? How could these barriers be overcome? [Possible prompts]
  - Fears and/or concerns over side effects?
  - Unrealistic expectations amongst patients
- What do you think are the key facilitators to treatment adherence and persistence?
- What do you do in situations where patients are not adhering to treatment? What approaches do you take (if any)?

Service changes

- **[Secondary care]** Are other members of the service involved in managing patients on Bisphosphonates? How? What is their role, and how is it different to yours?
- **[Primary care]** Are other members of the primary care team involved in managing patients on Bisphosphonates? How? What is their role, and how is it different to yours?
- Have there been any changes in the way you prescribe/administer bisphosphonate treatments in the past two years? Or any planned? [Possible prompt]
- If so, what has accounted for these changes and how do you feel about this?
- Are you aware of any recent evidence or guidance that has led to changes in the way you prescribe/administer bisphosphonate treatments?

**Part 3 - Fragility fracture risk and evidence**

- To what extent do you think that the bisphosphonate treatments which you prescribe/administer are effective in reducing the risk of fragility fracture?
- Do you think some Bisphosphonates are better than others for preventing fragility fractures?
- How do you decide whether or not the Bisphosphonate medication is working? If it is not working, what do you do about this?
- How large a concern do you perceive osteoporosis and the risk of fragility fracture to be for your patient group? What do you think is the reason for this? [Possible prompt]
- If patients have co-morbidities, where does osteoporosis come on their list of priorities/ concerns?
- How well developed, in your view, is evidence related to medication/treatments to reduce the risk of fragility fracture due to osteoporosis?
- How much of a priority is managing osteoporosis in your practice/area of work? Why?

**Part 4 - Conclude**

- Are there any other issues related to the provision of bisphosphonate medicines/treatments that you think are relevant for our study?

Thank participant for input and discuss any questions arising
